# Evaluation of Cervical Cerclage Timing and Perinatal Outcomes in Women with Cervical Insufficiency: A 10-Year Retrospective Study

**DOI:** 10.3390/jcm15020870

**Published:** 2026-01-21

**Authors:** Franciszek Ługowski, Julia Babińska, Kamil Jasak, Magdalena Litwińska, Ewelina Litwińska-Korcz, Zoulikha Jabiry-Zieniewicz, Artur Ludwin, Monika Szpotańska-Sikorska

**Affiliations:** 1 1st Department of Obstetrics and Gynaecology, Medical University of Warsaw, Starynkiewicza Sq. 1/3, 02-015 Warsaw, Poland; 2 Doctoral School, Medical University of Warsaw, 02-091 Warsaw, Poland

**Keywords:** cervical cerclage, cervical insufficiency, emergency cerclage, elective cerclage, perinatal outcomes

## Abstract

**Objective:** The objective was to evaluate the optimal timing of cervical cerclage insertion for perinatal outcomes, such as birthweight, gestational week, and pregnancy prolongation in women with diagnosed cervical insufficiency (CI). **Methods:** This retrospective study was conducted at the 1st Department of Obstetrics and Gynaecology of the Medical University of Warsaw, over a 10-year period. Maternal and perinatal outcomes were compared between 75 women divided into three groups based on the gestational week (GW) at cerclage insertion: (1) before 18 GW (n = 31), (2) 18–22 GW (n = 31), (3) after 22 GW (n = 13). Only single pregnancies were included in the final analysis in order to maintain the homogeneity of the population. The primary outcomes included the week of delivery and pregnancy prolongation following cervical cerclage insertion. Numerous secondary outcomes were also evaluated, including neonatal mortality, need for NICU hospitalization, Apgar score, birthweight, maternal white blood cell (WBC) count and C-reactive protein (CRP) levels. **Results:** Birth week was significantly associated with GW at insertion—35.8 ± 3.8 vs. 34.8 ± 5.2 vs. 32 ± 5.7, respectively, *p* = 0.016. Moreover, statistical difference was also found regarding birthweight of the analysed groups—2723.8 ± 951.6 g vs. 2518.5 ± 1167.9 g vs. 1886.7 ± 1011.2 g, respectively, *p* < 0.001, and pregnancy prolongation following cerclage insertion 20.4 ± 4.2 vs. 14.7 ± 5.5 vs. 7.3 ± 5.7 weeks, respectively, *p* < 0.001. **Conclusions:** Earlier cerclage placement (<18 weeks) is associated with significantly improved perinatal outcomes. However, this association largely reflects the benefit of prophylactic intervention over emergency ‘rescue’ procedures (common in the >22-week group). The sharp decline in outcomes after 22 weeks highlights the risks of advanced cervical dilation, suggesting that clinical management should prioritize risk assessment within the prophylactic window.

## 1. Introduction

Cervical insufficiency (CI) can be defined as dilatation and shortening of the cervix before the 37th week of gestation in the absence of preterm labour [1]. Most frequently, it is associated with premature rupture of the membranes, recurrent pregnancy loss, or preterm birth [1]. CI is estimated to occur in 8% of women with mid-trimester pregnancy losses and 1% of all pregnancies [2]. Interestingly, it is observed with a threefold higher frequency in black women [3]. The disease is caused by either structural or functional defects of the cervix [4]. Numerous risk factors for CI development have been identified, including collagen and elastin deficiency, surgical trauma to the cervix, a history of surgical procedures involving the vaginal portion of the cervix, premature cervical dilation, congenital uterine anomalies, multiple pregnancies, and intrauterine exposure to diethylstilbestrol (DES) [5,6]. Furthermore, new evidence emerges that inflammation and vaginal dysbiosis may impair the structural integrity of the cervix, thus contributing to CI [7]. On the molecular level, the concentrations of interleukin-6 (IL-6), IL-8, and monocyte chemoattractant protein-1 (MCP-1) in the amniotic fluid are thought to be increased in women with a short cervix prior to clinical manifestations, which could serve as an early predictive factor for the occurrence of preterm birth [8,9]. Currently, CI is defined as a measurement of <25 mm before 24 weeks of gestational age in singleton pregnancies with a history of pregnancy loss, preterm birth, or the presence of cervical changes detected by physical exam before 24 weeks on the transvaginal ultrasound examination or the presence of cervical changes detected before 24 weeks of gestation. The management of CI includes cervical cerclages, vaginal progesterone, and cervical pessaries [10]. Cervical cerclages strengthen the cervix, allowing it to maintain its length and preserve the mucus plug at the cervical opening, which protects against ascending infections [11]. However, the most optimal timing of cervical cerclage insertion is still a topic of debate [12]. Current international guidelines provide clear recommendations regarding the timing of cerclage: history-indicated procedures are typically performed between 12 and 14 weeks of gestation, and both ultrasound- and examination-indicated cerclages are recommended before 24 weeks [13]. What remains less clearly defined is whether the exact week of insertion within this guideline-approved window influences perinatal outcomes. Most existing studies compare elective versus emergency cerclage, without considering gestational age subcategories. Therefore, our study sought to refine the understanding of cerclage timing by stratifying women into clinically relevant gestational age groups (<18, 18–22, and >22 weeks), in order to explore whether earlier placement within the accepted window confers measurable benefit. While current guidelines recommend cervical cerclage placement before 24 weeks of gestation, few studies have investigated whether the exact week of insertion within this clinically accepted window affects perinatal outcomes. Our study, therefore, aimed not to redefine the general timing of cerclage, but to refine the understanding of optimal gestational age subranges within the established <24 week period.

In this study, we analysed the clinical data of patients with CI, in whom cervical cerclages were inserted at the 1st Department of Obstetrics and Gynaecology of the Medical University of Warsaw from January 2015 to February 2024. We aimed to establish the optimal timing of cerclage insertion based on the clinical characteristics, pregnancy, and neonatal outcomes of CI patients.

## 2. Materials and Methods

This investigation employed a retrospective cohort design and included women who underwent cervical cerclage at the 1st Department of Obstetrics and Gynaecology, Medical University of Warsaw, between 1 January 2015, and 29 February 2024. Ethical approval was obtained from the institutional review board (AKBE/345/2024). Clinical information was extracted from medical records, including maternal demographics (age, gravidity, parity), obstetric history (previous cervical insufficiency or cerclage), laboratory markers (WBC, CRP, PCT), gestational age at diagnosis and cerclage placement, and delivery outcomes. Neonatal data comprised Apgar scores, birthweight, need for NICU admission, and use of respiratory support (mechanical ventilation or CPAP). Written informed consent was obtained from all participants.

To ensure a uniform study population, only singleton pregnancies were included. Multiple gestations and women who delivered outside the study hospital were excluded, as complete perinatal data could not be reliably retrieved in such cases.

Participants were counselled regarding both conservative management and surgical intervention for asymptomatic cervical shortening or dilatation. For the purposes of analysis, women were stratified according to the gestational age at cerclage placement. While our previous analysis of this cohort focused on the clinical indication (elective vs. emergency) [14], the current study aims to evaluate the impact of gestational age at insertion as stratifying patients into three clinically distinct gestational age subgroups (<18, 18–22, and >22 weeks). We stratified by timing to determine if earlier intervention within these standard indication windows confers specific benefits, independent of the broad ‘elective’ or ‘emergency’ labels. In general, earlier procedures most likely corresponded to history- or ultrasound-based indications, whereas insertions beyond 22 weeks were typically performed as emergency interventions for cervical dilatation. Precise allocation by indication, however, was not possible.

The gestational age categories (<18, 18–22, >22 weeks) were selected to reflect clinically distinct timeframes rather than arbitrary intervals. <18 weeks corresponds to an early window in which cerclage is most often prophylactic, 18–22 weeks encompasses the typical period for ultrasound-indicated intervention prior to viability, and >22 weeks largely represents emergency/rescue placement. Although the indication type could not be determined for every case, these intervals align with major guideline recommendations and the natural progression of cervical insufficiency. Importantly, the choice of these gestational age intervals was not arbitrary but grounded in clinical practice. Cerclage performed before 18 weeks generally reflects history- or ultrasound-indicated prophylaxis, whereas 18–22 weeks represents the period when cervical shortening is most often detected on screening prior to viability. Interventions after 22 weeks, although technically feasible, largely represent emergency or rescue procedures in the presence of cervical dilation and bulging membranes. Although our dataset did not allow consistent classification by indication type, grouping by gestational age allowed us to approximate these distinct clinical contexts and analyse outcomes accordingly.

Experienced obstetricians at our tertiary institution performed singular and double cerclages using the McDonald, Wurm-Hefner, and Hervet’s techniques.

Indications for cervical cerclage were determined in accordance with the recommendations of the Polish Society of Gynecologists and Obstetricians for the management of a short cervix [15]. The decision to perform cerclage was based on the clinical presentation and gestational age at diagnosis. History-indicated cerclage was performed between 12 and 14 weeks of gestation in women with a history of second-trimester pregnancy loss due to painless cervical dilation or prior cerclage placement. Ultrasound-indicated cerclage was considered between 16 and 24 weeks of gestation in asymptomatic women with a trans- vaginally measured cervical length of <25 mm before 24 weeks of gestation, in the absence of uterine contractions or membrane rupture. Emergency (rescue) cerclage was performed before 26 weeks of gestation in women presenting with painless cervical dilation and visible or bulging fetal membranes, after excluding signs of intraamniotic infection or active labor.

Although many of these patients had a clinical history consistent with CI, they had not been referred for timely prophylactic intervention due to delayed presentation, insufficient medical documentation, or ambiguous risk profiles. In such instances, the decision to proceed with cerclage was driven primarily by real-time clinical assessment rather than historical risk factors.

In selected cases, a cervical pessary was employed during follow-up as an adjunct to cerclage, particularly in the presence of progressive cervical shortening or funneling in the second or early third trimester. Pessary placement was not performed at the time of cerclage insertion but was guided by individualized clinical judgment during surveillance.

Postoperative management was standardized across both groups. Vaginal micronized progesterone (200 mg daily) was administered in selected cases and continued until 36 weeks’ gestation or delivery, in accordance with the recommendations of the Polish Society of Gynecologists and Obstetricians [15]. Magnesium sulfate was administered specifically for fetal neuroprotection in patients at risk of imminent preterm birth before 34 weeks, rather than for tocolysis (for which Atosiban or Calcium Channel Blockers are utilized if indicated). Vaginal micronized progesterone was chosen over intramuscular injections, in line with current evidence supporting the vaginal route for cervical shortening.

Antenatal corticosteroids (24 mg of betamethasone or dexamethasone administered intramuscularly in divided doses) were given to women at risk of imminent preterm delivery between 24 + 0 and 34 + 0 weeks’ gestation, in accordance with national guidelines. Empirical antibiotic therapy was initiated perioperatively, particularly in emergency cases with exposed membranes or suspected subclinical infection. Antibiotic regimens were tailored by the managing clinicians in line with institutional protocols. In cases of bulging membranes or sonographic evidence of intraamniotic sludge, a 7-day regimen comprising ceftriaxone (1 g IV every 24 h), clarithromycin (500 mg orally every 12 h), and metronidazole (500 mg IV every 8 h) was employed [16].

Furthermore, due to the retrospective design and the long observation period, universal pre-operative urogenital cultures and granular data regarding specific antibiotic regimens (type, dosage, duration) or tocolytic use were not consistently available for the entire cohort. Therefore, the potential impact of the infectious milieu and variations in pharmacological management could not be assessed as confounding variables

The primary outcomes were gestational age at delivery and pregnancy prolongation following cerclage placement. Secondary outcomes comprised neonatal mortality, NICU admission, Apgar scores, birthweight, and maternal inflammatory markers, including white blood cell (WBC) count and C-reactive protein (CRP) levels. For maternal parameters, the peak WBC and CRP values documented during the hospitalization for cerclage insertion were assessed as surrogate markers of inflammation or subclinical infection. Because serial measurements and microbiological data were inconsistently available across the cohort, these variables were not incorporated into the final analysis.

Congenital infections were defined as clinical or laboratory evidence of neonatal infection identified within the first 72 h of life, suggestive of in utero transmission. This included early-onset neonatal sepsis characterized by clinical signs such as respiratory distress or temperature instability, accompanied by elevated inflammatory markers. Owing to the retrospective nature of the study, data regarding specific pathogens or culture-confirmed infections were limited.

### Statistical Analyses

Data were recorded in Microsoft Excel (version 14.0) and analyzed using IBM SPSS software (version 29.0; Armonk, NY, USA).

Variables were assessed for normality using the Shapiro–Wilk test. Due to non-normal distributions in key outcomes, non-parametric tests (Mann–Whitney U and Kruskal–Wallis) were employed for hypothesis testing. However, continuous variables are presented as Mean ± Standard Deviation (SD) to maintain consistency with previous literature and facilitate direct comparison. Moreover, categorical variables are expressed as counts and percentages. In cases where expected cell counts were low (n < 5), such as neonatal mortality or stillbirth, Fisher’s exact test was employed to ensure statistical validity. A *p*-value < 0.05 was considered indicative of statistical significance.

## 3. Results

Between 1 January 2015, and 29 February, 114 patients were diagnosed with CI in our department and underwent the procedure of cervical cerclage insertion, of whom 34 were excluded from further analysis due to incomplete perinatal data, and 5 due to twin pregnancies. Exclusions were strictly applied to ensure data homogeneity; patients with multiple gestations, delivery outside the study center, or incomplete medical records were removed. Consequently, the final study group (n = 75) represents a highly specific sub-cohort of singleton pregnancies with comprehensive follow-up data. The final analysis included 75 patients with singleton pregnancies. The women included in the analysis had a mean age of 32.8 years (range 20–42, SD ± 5.2). The mean gravidity was 3.07 (range 1–9, SD ± 1.8) and the mean parity was 1.79 (range 1–5, SD ± 1.0). The average gestational age at diagnosis was 18.6 weeks (range 12–31, SD ± 3.7), and the mean gestational age at the time of cerclage placement was 18.8 weeks (range 12–31, SD ± 3.6). Following the procedure, the mean gestational age at delivery was 34.7 weeks (range 24–41, SD ± 4.9), corresponding to an average pregnancy prolongation of 16.0 weeks (range 1–26, SD ± 6.8). In 42 cases, a cervical pessary was subsequently introduced because of progressive cervical shortening. Owing to variability in both the indication for placement and the timing of insertion, women who received a combined cerclage and pessary were not evaluated as a separate subgroup. All patients were hospitalized for the procedure in accordance with departmental protocol, typically for a duration of 2–3 days for observation post-insertion.

The study participants were further divided into three groups based on the GW at insertion: (1) cerclage insertion before 18 weeks of gestation, (2) insertion from 18 to 22 weeks of gestation, and (3) insertion after 22 weeks of gestation. The pregnancy outcomes of the three groups are compared in Table 1.

**Figure 1:**
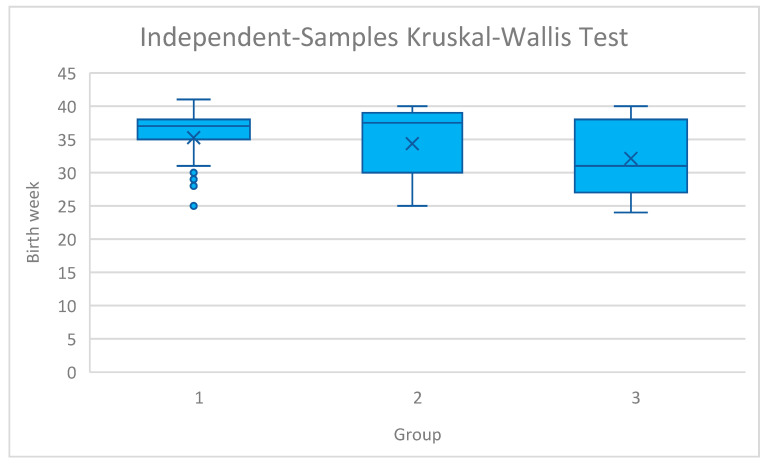
The distribution of birth weeks across study groups. Group 1: n = 31; Group 2: n = 31; Group 3: n = 13. 1—cerclage insertion before 18 weeks of gestation, 2—insertion from 18 to 22 weeks of gestation, 3—insertion after 22 weeks of gestation.

Regarding the primary outcomes, gestational age at delivery differed significantly across the cohorts (*p* = 0.016). Post hoc pairwise analysis revealed that this difference was primarily driven by the comparison between the early intervention groups and the late insertion group. Women in Group 1 delivered significantly later than those in Group 3 (*p* < 0.05). However, the difference in delivery timing between Group 1 (<18 weeks) and Group 2 (18–22 weeks) did not reach statistical significance, suggesting a plateau of benefit within the prophylactic window. An analysis of birth week distribution across the study groups is shown in Figure 1. In addition, the group with the latest cervical cerclage insertion was associated with the highest risk of delivery before 28 weeks of gestation (3% vs. 13% vs. 31%, respectively, *p* = 0.028). On the contrary, deliveries between 33 and 38 weeks of gestation were the most frequent in the early insertion group (52% vs. 19% vs. 8%, *p* = 0.013). No significant differences were found regarding delivery between 28 and 32 weeks of gestation (16% vs. 19% vs. 31%, *p* = 0.472) and after 38 weeks of gestation (35% vs. 39% vs. 23%, *p* = 0.687).

**Figure 2:**
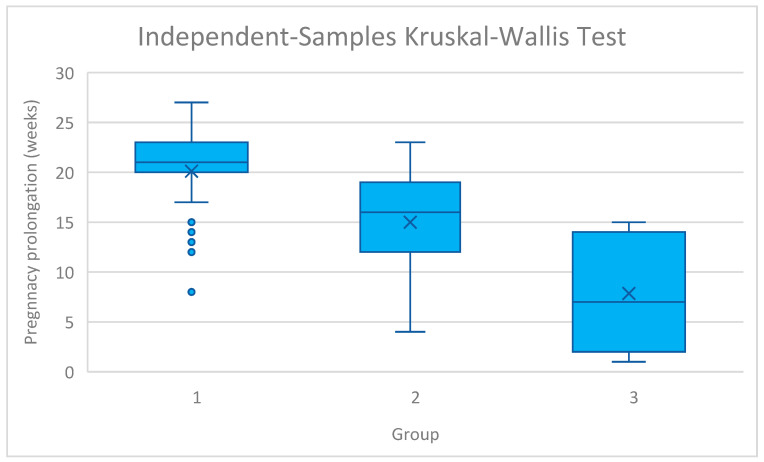
The distribution of pregnancy prolongation across study groups. Group 1: n = 31; Group 2: n = 31; Group 3: n = 13. 1—cerclage insertion before 18 weeks of gestation, 2—insertion from 18 to 22 weeks of gestation, 3—insertion after 22 weeks of gestation.

Moreover, birthweight showed a similar pattern (2723.8 ± 951.6 g in group 1 vs. 2518.5 ± 1167.9 g in group 2 vs. 1886.7 ± 1011.2 g in group 3, *p* < 0.001). However, no significant differences were revealed in pairwise comparisons between the study cohorts regarding birthweight. In addition, pregnancy prolongation was significantly longer in group 1 compared with the other groups (20.4 ± 4.2 weeks vs. 14.7 ± 5.5 weeks vs. 7.3 ± 5.7 weeks, *p* < 0.001). Pregnancy prolongation across the study groups was analysed in Figure 2. All pairwise comparisons for pregnancy prolongation were statistically significant after Bonferroni correction. Group 1 had significantly longer pregnancy prolongation than both Group 2 and Group 3, and Group 2 had significantly longer prolongation than Group 3.

Numerous secondary outcomes were also analysed in our study. Regarding neonatal outcomes, no significant differences were found for the NICU admission rates (13% vs. 10% vs. 38%, *p* = 0.036), CPAP requirement (6% vs. 16% vs. 31%, *p* = 0.079), and the need for intubation (3% vs. 10% vs. 23%, *p* = 0.480). Furthermore, Apgar scores did not differ significantly between the groups—at 1 min (9.2 ± 1.5 vs. 8.3 ± 3.0 vs. 7.9 ± 2.2, *p* = 0.061), 5 min (9.3 ± 1.6 vs. 8.8 ± 2.4 vs. 8.6 ± 1.7, *p* = 0.101), and 10 min (9.6 ± 0.8 vs. 8.9 ± 2.4 vs. 9.0 ± 1.4, *p* = 0.208). No statistically significant differences were also found regarding the neonatal death (0% vs. 6% vs. 0%, *p* = 0.326), stillbirth (0% vs. 6% vs. 0%, *p* = 0.284), and the congenital infection rate (3% vs. 6% vs. 31%, *p* = 0.078).

The analysis of maternal outcomes revealed no significant differences in CRP levels (7.2 ± 5.9 vs. 11.4 ± 16.4 vs. 15.5 ± 18.0 mg/L, *p* = 0.449). However, peak maternal WBC was the highest in group 3 (10.4 ± 3.8 vs. 11.1 ± 3.3 vs. 13.7 ± 2.8 G/L, *p* < 0.001). Of importance, a statistically significant difference was recorded for the pre-birth MgSO4 administration rates (3% vs. 19% vs. 31%, *p* = 0.033).

## 4. Discussion

Cervical cerclages remain an important obstetric intervention for women with CI as they have been proven to reduce the rate of preterm birth, therefore improving perinatal outcomes [17].

The timing of cervical cerclage insertions varies depending on the recommendations and indications. According to the Royal College of Obstetricians and Gynaecologists (RCOG), a history-indicated cervical cerclage should be considered between 11 and 14 weeks of gestation, ultrasound-indicated cerclages before 24 weeks of gestation, whereas rescue cerclages, up to 27 + 6 weeks [13]. Furthermore, the International Federation of Gynaecology and Obstetrics (FIGO) recommends the same timing for ultrasound-indicated cerclages and the insertion of emergency cerclages before 24 weeks of gestation [18]. However, the guideline does not discuss the optimal timing of history-indicated cervical cerclages [18].

The aim of our study was to determine if earlier placement of cervical cerclages is associated with better perinatal outcomes. Our analysis revealed that earlier placement of cervical cerclages, especially before 18 weeks of gestation, results in higher mean birth week and longer pregnancy prolongation. However, it is crucial to recognize that gestational age at insertion in this study serves as a proxy for clinical status. The cohort treated >22 weeks (Group 3) predominantly represents ‘rescue’ cerclages performed in the setting of advanced cervical dilation. Therefore, the observed decline in outcomes is likely driven by the severity of CI at the time of intervention, rather than the calendar timing alone. This supports the ‘pathophysiological lead time’ hypothesis, suggesting that intervention is most effective before the inflammatory cascade associated with overt cervical shortening and membrane exposure is fully established. Consequently, the statistical benefit of early timing reflects the distinct advantage of prophylactic management over emergency salvage in patients with advanced cervical instability. This interpretation is further supported by Armarnik et al., who demonstrated that pregnancy prolongation and neonatal outcomes are strongly influenced by the degree of cervical dilation and clinical presentation at the time of insertion, rather than gestational age alone [19]. Their findings reinforce that the baseline cervical pathology is the primary driver of outcomes in late rescue procedures [19].

The ‘earlier is better’ trend reflects the benefit of prophylactic intervention (Groups 1 and 2) over emergency salvage (Group 3). Moreover, the results of our study indicate a general association between the week of cervical cerclage insertion and birthweight. However, no significant difference was noted in pairwise comparisons. Of importance, the variability of birthweight, birth week, and pregnancy prolongation increased with later cervical cerclage placement (Figure 1 and Figure 2). Thus, earlier insertion appears to correlate with greater predictability of pregnancy outcomes.

There is scarce literature data regarding the optimal timing of cervical cerclage insertion for CI management, as previous studies largely focused on the comparison of emergency vs. elective cerclages [14,20]. International consensus documents, including the RCOG Green-Top Guideline No. 75 and FIGO Good Practice Recommendations, emphasize that history-indicated cerclage should be placed at 12–14 weeks, and that ultrasound- or examination-indicated cerclage should be placed before 24 weeks [13,18]. Our findings are therefore not intended to challenge these recommendations, but rather to refine the timing of intervention within this already accepted therapeutic window. Specifically, our results suggest that insertion before 18 weeks is associated with more favourable outcomes, including longer pregnancy prolongation and higher gestational age at delivery, compared with later insertion. Incoherently with our study, He et al. found no significant difference regarding the pregnancy prolongation between early (insertion at 14–18 GW) and middle (insertions at 19–27 GW) groups [12]. On the contrary, the same study reported a significantly higher rate of term births in the early group compared to the late insertion cohort [12]. In addition, a study by Hollis et al. aimed to compare the pregnancy outcomes between patients with cervical cerclage insertion before and after 13 weeks of gestational age. In that study, no significant differences were found regarding gestational age at delivery, the rate of births before 37 and 34 weeks of gestation [21]. Furthermore, numerous perinatal outcomes were assessed in a study by Chen et al., which divided the analysed population into 3 cohorts based on the indication for cervical cerclage. The subgroups differed significantly concerning mean GW at cerclage insertion (history-indicated—15.7 weeks, ultrasound-indicated—19.1 weeks, and physical examination-indicated—23.6 weeks) [22]. Consistent with our analysis, significant differences were reported for GW at delivery, with the longest duration of pregnancies in the early insertion cohort. Likewise, pregnancy prolongation was significantly longer in the first group. Moreover, deliveries before 28 weeks of gestation were significantly more frequent in the third group, similarly to our results. Consistent with the findings of our study, the highest mean birthweight was observed in the first group. On the contrary, Chen et al. reported significant differences regarding Apgar scores, which our study did not confirm. In accordance with our study, a significant association between the GW at cerclage insertion and birth week was also observed by Chan et al., who additionally found a longer prolongation of pregnancy and higher neonatal birthweight in the group of the earliest cerclage insertion [23].

Importantly, our study also contextualizes the significance of cerclage timing within the broader clinical picture, highlighting a dose–response relationship between gestational age at insertion and pivotal neonatal outcomes. While the benefits of early cerclage were most pronounced before 18 weeks, a gradual decline in birthweight and gestational age at delivery was evident as cerclage was delayed beyond this point. This trend suggests a temporal therapeutic window during which cerclage exerts maximum benefit, potentially aligned with the early second trimester, when cervical shortening may first become detectable via transvaginal ultrasonography in high-risk populations.

This assertion is supported by the FIGO and RCOG guidelines, which emphasize cerclage placement prior to 24 weeks of gestation for both ultrasound- and examination-indicated cases [13,18]. Nonetheless, our data raise the possibility that even within this recommended window, earlier intervention—especially in the presence of prior obstetric history or subtle cervical changes—may confer additional benefit. This is consistent with the “pathophysiological lead time” hypothesis, wherein subclinical cervical remodeling and biochemical alterations, such as upregulated inflammatory cytokines (e.g., IL-6, monocyte chemoattractant protein-1), precede overt cervical shortening [8,9]. Early cerclage may therefore mitigate these changes before they manifest structurally.

Interestingly, while the differences in NICU admission rates, need for mechanical ventilation, and neonatal mortality were not statistically significant across groups, trends were noted favouring earlier cerclage placement. The lack of statistical significance may be attributable to the modest sample size of our cohort, particularly in the subgroup undergoing late cerclage insertion. Nonetheless, the observed differences in gestational age and birthweight likely translate into meaningful clinical benefit in terms of neonatal morbidity, warranting further investigation in larger prospective cohorts.

Furthermore, while maternal inflammatory markers such as CRP did not differ significantly, elevated WBC counts in the late cerclage group raise questions about subclinical infection or sterile inflammation contributing to cervical shortening in these cases. This supports the growing body of literature emphasizing the role of inflammation in the pathogenesis of CI, and suggests that earlier intervention may also help avoid downstream inflammatory cascades associated with adverse outcomes.

In conclusion, our findings reinforce the principle that earlier cerclage placement, particularly prior to 18 weeks of gestation, is associated with improved perinatal outcomes in women with cervical insufficiency. These results contribute meaningfully to the ongoing refinement of clinical decision-making in this domain and suggest that the gestational age at cerclage insertion should be a critical consideration when formulating individualized treatment plans. Future prospective studies, ideally randomized where feasible, are warranted to further delineate the optimal window for cerclage placement and to explore the molecular underpinnings of cervical remodelling in early pregnancy.

Notably, our results complement our earlier analysis of elective versus emergency cerclage [14]. While that study highlighted differences based on indication, the present analysis provides additional granularity by examining gestational age at insertion irrespective of indication. This distinction is important, as timing may independently influence pregnancy outcomes, even when indication and surgical technique overlap. The consistency of findings across both analyses strengthens the conclusion that earlier intervention is associated with improved perinatal outcomes.

This study has several limitations that must be acknowledged. First, the relatively small sample size, particularly within the subgroup of patients who underwent cerclage after 22 weeks of gestation, limits the statistical power and generalizability of the findings. The reduced sample size in this cohort may have also contributed to the lack of statistical significance observed in certain neonatal outcomes, such as NICU admission rates and respiratory support requirements, despite evident trends. Another important limitation of this study is the heterogeneity in both the indication and clinical context of cervical cerclage placement. While patients were grouped according to gestational age at insertion, the underlying reasons for cerclage varied significantly, ranging from elective, history-indicated cases to urgent, rescue procedures performed in the setting of cervical dilation and bulging membranes. These distinct clinical scenarios reflect different stages of cervical insufficiency and potentially divergent underlying pathophysiology, which may influence outcomes independently of timing. As a result, the comparison between groups should be interpreted with caution, as differences in perinatal outcomes may not be attributable to timing alone, but also to the clinical circumstances necessitating cerclage. A further limitation is the absence of an a priori sample size calculation. Additionally, the retrospective design of the study constitutes another limitation. Future prospective studies with standardized protocols and larger populations are warranted to validate these findings and refine clinical recommendations regarding the optimal timing of cervical cerclage placement.

## 5. Conclusions

Within the timeframe of viability, earlier cerclage placement is associated with significantly improved perinatal outcomes. However, this association likely reflects the superior efficacy of prophylactic intervention over emergency ‘rescue’ procedures, highlighting that the window of opportunity closes as structural cervical failure advances.

The sharp decline in outcomes observed after 22 weeks highlights the risks associated with advanced cervical dilation and the importance of intervening before structural failure is irreversible. Therefore, clinical management should prioritize early risk assessment and timely placement of cerclage within the prophylactic window to maximize neonatal survival and reduce the morbidity associated with late, emergency interventions.

## Figures and Tables

**Table 1:** Comparison of perinatal outcomes depending on the week of cervical cerclage insertion.

	<18 GW (n = 31)	18-22 GW (n = 31)	>22 GW (n = 13)	*p*
Maternal age, (mean ± SD), years	33.7 ± 4.6	32.8 ± 6.0	30.4 ± 4.5	0.109
Birth week (mean ± SD)	35.8 ± 3.8	34.8 ± 5.2	32.0 ± 5.7	0.016
GW at diagnosis (mean ± SD)	15.3 ± 1.2	19.6 ± 1.7	24.4 ± 2.6	<0.001
GW at cerclage insertion (mean ± SD)	16.2 ± 1.1	19.8 ± 1.4	24.5 ± 1.7	<0.001
Numer of pregnancies (mean ± SD)	3.8 ± 2.0	2.6 ± 1.5	2.5 ± 1.7	0.002
Number of delivery (mean ± SD)	2.2 ±1.2	1.5 ± 0.7	1.5 ± 1.1	0.024
Cesarean section	15 (48%)	18 (58%)	9 (69%)	0.09
PPROM	3 (10%)	5 (16%)	1 (8%)	0.667
Progesterone administration	28 (90%)	25 (81%)	9 (69%)	0.102
Birthweight (mean ± SD), g	2723.8 ± 951.6	2581.5 ± 1167.9	1886.7 ± 1011.2	<0.001
Need for NICU hospitalization	4 (13%)	3 (10%)	5 (38%)	0.036
Need for CPAP	2 (6%)	5 (16%)	4 (31%)	0.079
Need for intubation	1 (3%)	3 (10%)	3 (23%)	0.480
Neonatal death	0 (0%)	2 (6%)	0 (0%)	0.326
Stillbirth	0 (0%)	2 (6%)	0 (0%)	0.284
Apgar at 1 min (mean ± SD)	9.2 ± 1.5	8.3 ± 3	7.9 ± 2.2	0.061
Apgar at 5 min (mean ± SD)	9.3 ± 1.6	8.8 ± 2.4	8.6 ± 1.7	0.101
Apgar at 10 min (mean ± SD)	9.6 ± 0.8	8.9 ± 2.4	9 ± 1.4	0.208
Congenital infection	1 (3%)	2 (6%)	4 (31%)	0.078
Highest maternal WBC during hospitalization (mean ± SD), G/L	10.4 ±3.8	11.1 ± 3.3	13.7 ± 2.8	<0.001
Highest maternal CRP during hospitalization (mean ± SD), mg/L	7.2 ±5.9	11.4 ± 16.4	15.5 ± 18.0	0.449
Pre-birth MgSO4	1 (3%)	6 (19%)	4 (31%)	0.033
Gestational week at cerclage removal (mean ± SD)	36.4 ± 0.9	32.2 ± 4.5	34 ± 5.0	0.087
Cervical pessary insertion	17 (55%)	18 (58%)	8 (62%)	0.828
Pregnancy prolongation following cervical cerclage insertion (mean ± SD). weeks	20.4 ± 4.2	14.7 ± 5.5	7.3 ± 5.7	<0.001
Delivery before 28 weeks of gestation	1 (3%)	4 (13%)	4 (31%)	0.028
Delivery between 28 and 32 weeks of gestation	5 (16%)	6 (19%)	4 (31%)	0.472
Delivery between 33 and 38 weeks of gestation	16 (52%)	6 (19%)	1(8%)	0.013

## Data Availability

Data are available from the corresponding author upon reasonable request.
